# High-throughput sequencing dataset of raw and treated wastewater from Jeddah, Saudi Arabia

**DOI:** 10.1016/j.dib.2026.113174

**Published:** 2026-08-25

**Authors:** Manish P. Victor, Nawaf Al Shehri, Didrik H. Grevskott, Walied M. Alarif, Aasim M. Ali, Nachiket P. Marathe

**Affiliations:** a Department of Contaminants and Biohazards, Institute of Marine Research (IMR), Bergen, Norway; b Marine Chemistry Department, Faculty of Marine Sciences, King Abdulaziz University, P.O Box 80207, Jeddah, 21589, Saudi Arabia

**Keywords:** Wastewater, Metagenomics, Bacterial diversity, Antibiotic resistance genes, Biocide resistance, Metal resistance

## Abstract

Wastewater is a major point source of pollution and antimicrobial resistance (AMR) in the environment. The aim of this study was to determine the microbiota and antimicrobial resistance genes present in both the influent and effluent from a biological wastewater treatment plant situated in Jeddah Second Industrial City, Saudi Arabia, using metagenomics. Water samples were collected, and the DNA extracted from these samples was subjected to metagenomic sequencing (one influent and one effluent) using Illumina NovaSeq. Our analysis yielded ∼88 Gigabases. The predominant bacterial genera identified were Actinomycetota, Psuedomonadota, and Bacillota, and β-lactamases emerged as the most common class of resistance genes. Eventhough these are unreplicated single observations they are expected to be a valuable resource for researchers focusing on wastewater studies, especially in the middle East, offering insights into bacterial diversity and the spectrum of resistomes associated with these microbial communities.

Specifications Table

**Table:** 

Subject	Biology
Specific subject area	Microbiology and genomics
Type of data	Shotgun metagenomic sequencing data
Data collection	Wastewater grab samples were collected on the 10th of November 2022, from Jeddah Second Industrial City, KSA (21.3997715N, 39.2291861E). They were collected in HDPE bottles, kept on ice during transport, and stored at <-20°C until shipment. For the flight, they were again placed in an icebox and remained frozen until their arrival in Bergen, Norway.
Data source location	Latitude: 21.3997715° N; 39.2291861°E; Jeddah, Saudi Arabia
Data accessibility	Raw sequencing data were deposited in the Sequence Read Archive (SRA) under BioProject accession no. PRJNA1424426 and is publicly available under the accession numbers SRR (effluent: SRR37263313, influent: SRR37263314). https://www.ncbi.nlm.nih.gov/bioproject/PRJNA1424426?report=docsum
Related research article	None

## 1 Value of the Data

• These data shed light on the largely unknown microbial diversity in treated and untreated wastewater from Jeddah city.
• This information is valuable for scientists studying AMR in wastewater of Saudi Arabia and elsewhere, environmental engineers and public health authorities in the Gulf region but also worldwide.
• This data provides essential insights for monitoring the dissemination of resistance genes into the environment in the middle east

## 2 Background

The examination of wastewater, encompassing both influent and effluent, is of paramount importance because of its role as a reservoir and a conduit for antibiotic resistant bacteria (ARB) and antibiotic resistance genes (ARGs). The presence of bacteria such as ESKAPE (*Enterococcus faecium, Staphylococcus aureus, Klebsiella pneumoniae, Acinetobacter baumannii, Pseudomonas aeruginosa,* and *Enterobacter* spp.) in wastewater presents significant public health challenges owing to their multidrug resistance capabilities [1,2]. Wastewater originating from hospitals, agriculture, and other anthropogenic sources serves as a transitional medium for the dissemination of ARB and ARGs into the environment [3,4]. The persistence and proliferation of ARB and ARGs in wastewater treatment settings typically result from inefficient removal processes in conventional treatment plants. Standard procedures involving UV disinfection and chlorine exhibit limited efficacy in eliminating ARGs, prompting a shift towards advanced oxidation processes (AOPs) and other innovative methods that could enhance the elimination of deleterious genes [5]. Biological treatments play a crucial role in wastewater management by employing microbial communities to decompose organic matter. However, these processes occasionally facilitate horizontal gene transfer between bacteria, thereby promoting the spread of ARGs [6]. This necessitates an additional focus on enhancing biological treatment frameworks to curb the propagation of resistance within WWTPs. Despite these challenges, biological treatment remains essential owing to its cost-effectiveness and capacity to efficiently manage large-scale wastewater purification. Understanding bacterial dynamics and resistance mechanisms in these water systems will aid in formulating improved treatment strategies and policies aimed at minimizing risks for dissemination of AMR into the environment.

## 3 Data Description

**Fig. 1:**
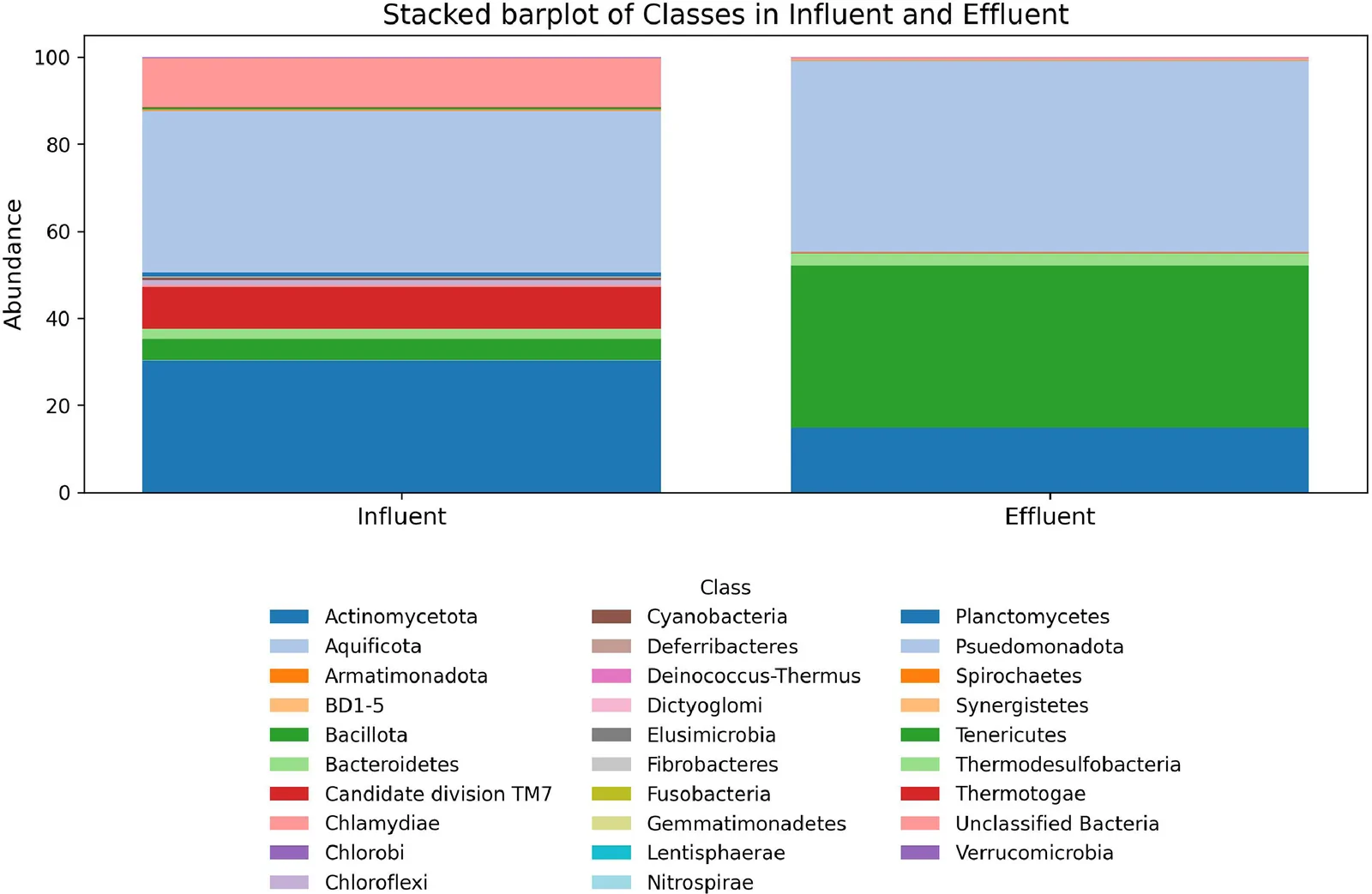
Bacterial (Phylum) diversity for the influent and effluent samples.

Approximately ∼88.1 Gigabases representing around 602 million reads (unreplicated single observations) from two wastewater samples (influent and effluent). Analysis of 16S rRNA gene reads revealed the presence of 26 phyla in the influent sample and 29 phyla in the effluent sample (Fig. 1). Based upon un-replicated single observations, in the influent sample on the class-level, Actinomycetota were the most prevalent at approximately 29%, followed by Pseudomonadota at approximately 26%. In contrast, the effluent sample was dominated by Pseudomonadota at approximately 38% and Bacillota at approximately 29%. Several genera including pathogens, including *Enterococcus, Staphylococcus, Klebsiella, Acinetobacter,* and *Pseudomonas*. The analysis reveals that sulfonamide resistance were the most abundant in the influent sample, followed by genes conferring resistance to aminoglycosides and rifamycin. In contrast, β-lactamases genes were present at comparatively low levels in effluent. In the effluent sample, efflux pump genes from the RND family were predominant, while β-lactamases genes remained at relatively minor levels (Supplementary Files). Various clinically important ARGs were detected in effluent samples. We detected resistance genes against last resort antibiotics like *tet*(X) variants, *mcr* genes, carbapenemases, *cfr* genes and vancomycin resistance genes in both influent and effluent. Many resistance genes, including extended spectrum β-lactamases identified in the samples are commonly found in ESKAPE pathogens, supporting the fact that wastewater is a significant pathway for clinically important genes reaching the environment [7]. Several efflux pumps encoding biocide resistance were observed under metal resistance genes in both the influent and effluent samples. Several virulence factors (VFs) related to capsule formation, adhesion, siderophore production, pilus formation, and toxin production were also identified. Clinically significant genes associated with toxin production, such as *toxA/B*, were detected in these samples. Among the mobile genetic elements (MGEs) studied, integron-associated integrases (*intI*) were identified in both the influent and effluent samples. The metabolic functions derived from these metagenomes indicated their active roles in methane metabolism, nutrient cycling and phenol degradation (Supplementary Files).

## 4 Experimental Design, Materials and Methods

Wastewater influent and effluent were collected using composite method in HDPE bottles from a treatment plant in Jeddah Second Industrial City, transported on ice, and frozen until analysis. The processing of wastewater samples was performed as previously described [8]. Briefly, from each sample, 30–50 mL was vacuum-filtered through S-Pak filters (pore size 0.22µm) (Millipore, USA), after which the filters were transferred to 50 mL Falcon tubes and stored at -20°C until further use. Genomic DNA was extracted from the filters using the DNeasyPowerWater kit (Qiagen, Germany) following the manufacturer’s protocol. The extracted DNA was quantified using a dsDNA BR (Broad-Range) assay kit on a Qubit™ 2.0 Fluorometer and NanoDrop™ 2000 Spectrophotometer (Thermo Scientific, USA). Sequencing libraries for shotgun metagenomics were prepared using the Nextera DNA Flex Library Prep kit (Illumina, USA) and sequencing was performed using the Illumina NovaSeq platform (Illumina, USA), with 2 × 150 bp chemistry on a S4 flow cell, at the Norwegian Sequencing Center (Oslo University Hospital,Ullevål, Oslo, Norway). The sequences were decontaminated of human DNA using bmtagger (https://github.com/movingpictures83/BMTagger) and quality-trimmed using Trim Galore v.0.6.10 (http://www.bioinformatics.babraham.ac.uk/projects/trim_galore/) with a quality score of 30 (Supplementary). The quality-processed reads from the metagenomes were mapped to a high-quality, manually curated database of unique ARGs (AMRFfinder, ResFinder, ARGannot and CARD) [[9], [10], [11]], biocide resistance genes (v 2.0) [12], metal resistance genes (v 2.0) [12], MGEs [13], and VFs [14], using usearch -usearch_global reads.fastq -db amr.udb -id 0.9 -blast6out hits.b6 -strand plus -maxaccepts 1 (USEARCH v11,64 bit) [15] as suggested by Marathe et al. (2017) [7]. For quantitative taxonomic distribution, quality-filtered shotgun reads were used to extract reads corresponding to the small subunit (SSU) 16S bacterial ribosomal RNA (rRNA) genes from metagenomes and were assigned to different taxonomic groups using Metaxa2 with all the default values for the parametrs. (v.2.2.3) [7,16]. The relative abundance of each taxon at each taxonomic level (such as phyla and class) was calculated by determining the percentage of 16S rRNA gene sequence counts from each metagenome. Fun4me (v 2.0) was used to determine the functional composition of microbial communities from the reads [17].

## Limitations

This study has several limitations that should be acknowledged. First, the lack of replication restricts the generalizability of the findings. Furthermore, the absence of detailed metadata on operational parameters of the treatment plants, such as hydraulic retention time (HRT), solids retention time (SRT), and specific treatment stages, limits the ability to fully interpret the results within the context of plant performance and process dynamics. Addressing these limitations in future research will be essential to strengthen the robustness and applicability of the conclusions drawn.

## Ethics Statement

The authors have read and follow the ethical requirements for publication in *Data in Brief* and confirming that the current work does not involve human subjects, animal experiments, or any data collected from social media platforms.

## CRediT Author Statement

Conceptualization: NPM. Data curation: MPV, NPM, DHG, NAS, AA. Formal analysis: MPV, NPM, DHG. Funding acquisition: NPM, AA. Investigation: NPM, MPV. Methodology: NPM, MPV, DHG. Project administration: NPM, AA. Resources: NPM. Software: MPV, NPM. Supervision: NPM. Validation: NPM, MPV, DHG, NAS, AA. Visualization: MPV, NPM. Writing – original draft: NPM, MPV. Writing – review & editing: NPM, MPV, DHG, NAS.

## Appendix Supplementary Materials

Image, image 1 dummy alt text

Image, image 2 dummy alt text

Image, image 3 dummy alt text

Image, image 4 dummy alt text

Image, application 5

Image, application 6

Image, application 7

Image, application 8

Image, application 9

Image, application 10

Image, application 11

Image, application 12

Image, application 13

Image, application 14

Image, application 15

Image, application 16
