# Supplementary material for: High-throughput sequencing dataset of raw and treated wastewater from Jeddah, Saudi Arabia
Source: Data Brief. 2026 Aug 25;68:113174. doi: 10.1016/j.dib.2026.113174 (PMC13579928; doi:10.1016/j.dib.2026.113174)

Nonpariel Curve : Influent Forward

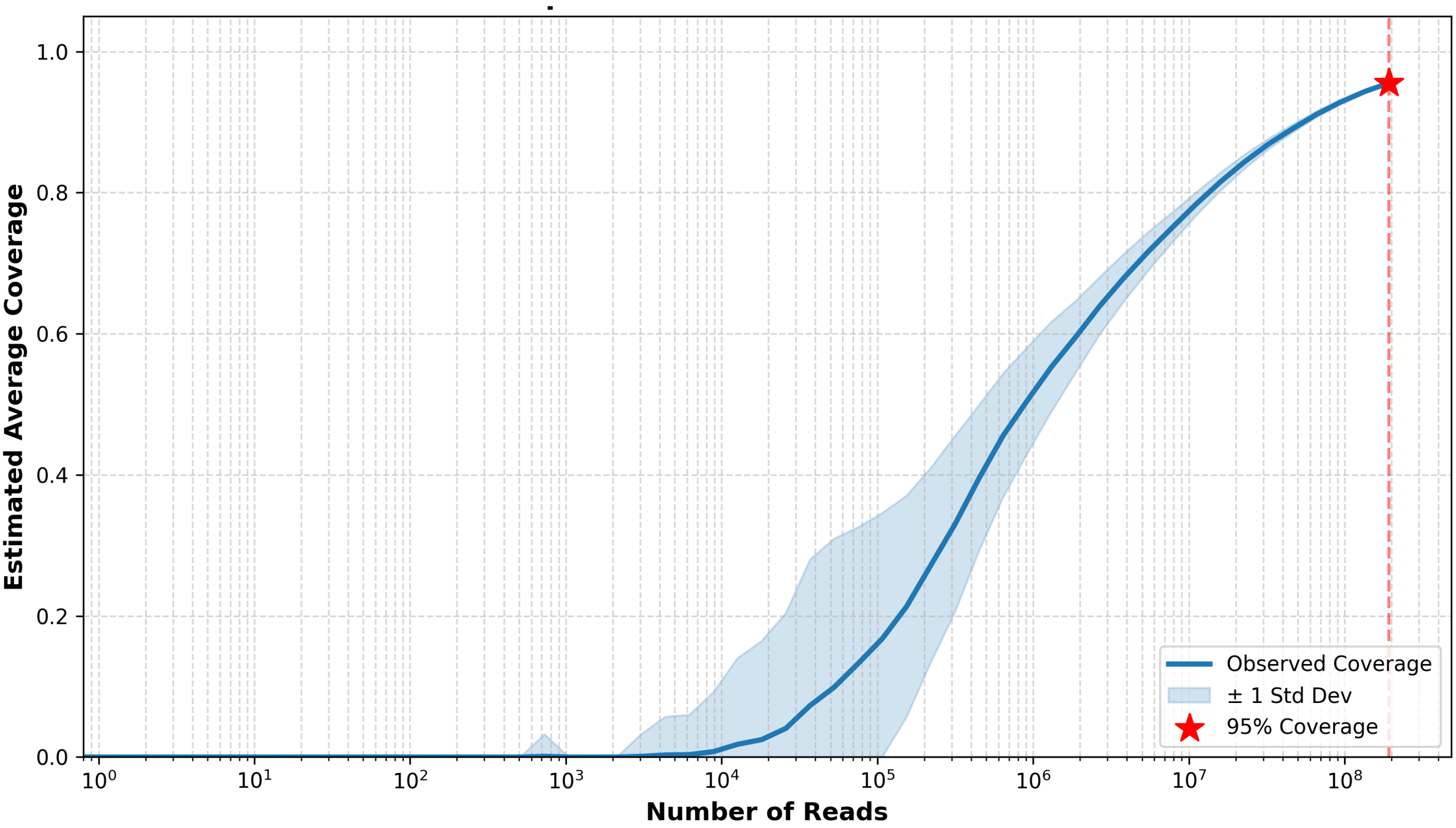

Supplement: Supplementary file 1 [file mmc1.pdf]
