# Supplementary material for: High-throughput sequencing dataset of raw and treated wastewater from Jeddah, Saudi Arabia
Source: Data Brief. 2026 Aug 25;68:113174. doi: 10.1016/j.dib.2026.113174 (PMC13579928; doi:10.1016/j.dib.2026.113174)
Supplement: Supplementary file 3 [file mmc3.pdf]

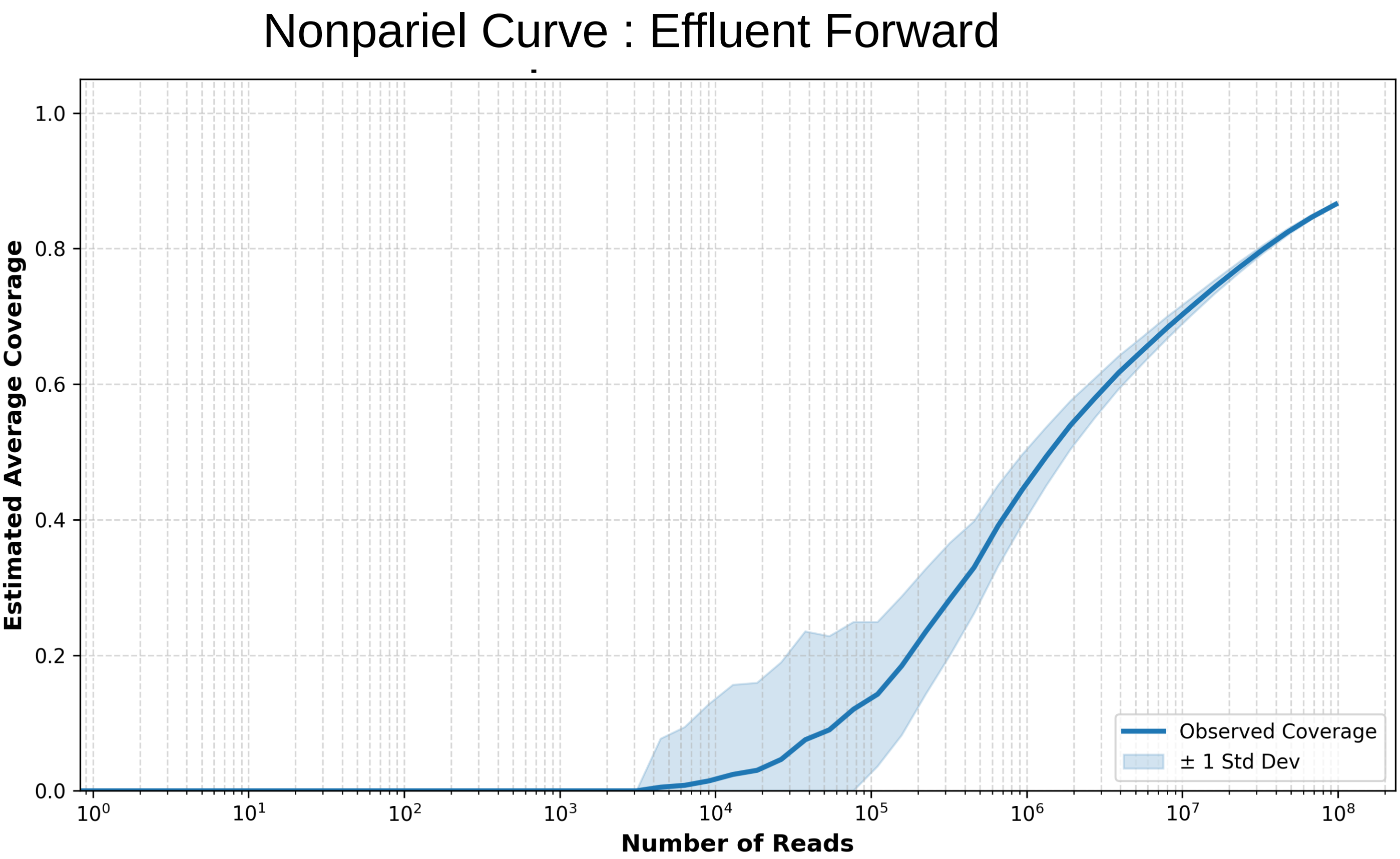

Nonpariel Curve : Effluent Forward
1.0
0.8
0.6
0.4
0.2
0.0
Estimated Average Coverage
10^0
10^1
10^2
10^3
10^4
10^5
10^6
10^7
10^8
Number of Reads
Observed Coverage
± 1 Std Dev
